# Neural circuits containing olfactory neurons are involved in the prepulse inhibition of the startle reflex in rats

**DOI:** 10.3389/fnbeh.2015.00074

**Published:** 2015-03-25

**Authors:** Haichen Niu, Xiaobin He, Ting Zhou, Xi Shi, Qiang Zhang, Zhijian Zhang, Yuehua Qiao, Fuqiang Xu, Min Hu

**Affiliations:** ^1^Department of Genetics, Xuzhou Medical CollegeXuzhou, China; ^2^The Institute of Audiology and Speech Science, Xuzhou Medical CollageXuzhou, China; ^3^Key Laboratory of Magnetic Resonance in Biological Systems and State Key Laboratory of Magnetic Resonance and Atomic and Molecular Physics, Wuhan Institute of Physics and Mathematics, Chinese Academy of SciencesWuhan, China; ^4^Wuhan National Laboratory for Optoelectronics, Huazhong University of Science and TechnologyWuhan, China; ^5^Department of Ophthalmology, The Second People’s Hospital of Yunnan ProvinceKunming, China

**Keywords:** olfactory dysfunction, sensory stimulus, prepulse inhibition (PPI), NMDA receptor, neural trace

## Abstract

Many neuropsychiatric disorders, such as schizophrenia, have been associated with olfactory dysfunction and abnormalities in the prepulse inhibition (PPI) response to a startle reflex. However, whether these two abnormalities could be related is unclear. The present investigations were designed to determine whether theblockage of olfactory sensory input by zinc sulfate infusion in the olfactory naris (0.5 ml, 0.17 M, ZnE) can disturb the PPI response. Furthermore, a bilateral microinjection of lidocaine/MK801 in the olfactory bulb (OB) was administered to examine whether the blockage of olfactory sensory input could impair the PPI response. To identify the neural projection between olfaction and PPI-related areas, trans-synaptic retrograde tracing with the recombinant pseudorabies virus (PRV) was used. Our results demonstrated that blockage of olfactory sensory input could disturb olfactory behavior. In the function studies, we demonstrated that blockage of olfactory sensory input could impair the pre-pulse inhibition of the startle response following decreased c-Fos expression in relevant brain regions during the PPI responses. Furthermore, similar and more robust findings indicated that blockage of olfactory sensory input by microinjection of lidocaine/MK801 in the OB could impair the PPI response. In the circuit-level studies, we demonstrated that trans-synaptic retrograde tracing with PRV exhibited a large portion of labeled neurons in several regions of the olfactory cortices from the pedunculopontine tegmental nucleus (PPTg). Thus, these data suggest that the olfactory system participates in the PPI regulating fields and plays a role in the pre-pulse inhibition of the startle response in rats.

## Introduction

An intact nervous system engages a variety of mechanisms to suppress or “filter out” irrelevant information in sensory, cognitive and motor domains to enable an individual to focus on the most salient stimuli in the environment (Braff and Geyer, 1990). The prepulse inhibition reflex (PPI) is a measure of the suppression of irrelevant information, which measures an individual’s decreased response to unexpected auditory stimuli, referred to as an acoustic startle response (ASR), when a weak auditory pulse precedes the unexpected stimuli (Braff and Geyer, 1990). Clinical studies have demonstrated that many neuropsychiatric disorders exhibit an impaired ability to suppress irrelevant information. For example, patients with schizophrenia exhibited impairments in automatically filtering irrelevant thoughts and sensory stimuli (Geyer et al., 2002). Clinical observations have also demonstrated that olfactory dysfunction occurred in neuropsychiatric disorders (Martzke et al., 1997). Furthermore, in rodents, some animal models of neuropsychiatric disease have been demonstrated to exhibit olfactory dysfunction (Huckins et al., 2013). Currently, whether there is a relationship between olfactory dysfunction and an abnormal PPI is unclear.

To explore the contribution of blockage of olfactory sensory input in the PPI response in rats, an animal model was established by naris infusion of zinc sulfate (ZnE) to create an olfactory blockage model (Alberts and Galef, 1971). The activities of the PPI-related cortex were evaluated by c-Fos expression, which was used to map the distribution of neurons activated by physiological and pharmacological stimuli (Sagar et al., 1988). Previous studies have indicated that the amygdala and hippocampus were part of the PPI-related neural circuit (Swerdlow et al., 2001). Furthermore, the pedunculopontine tegmental nucleus (PPTg) played an important role in the PPI response (Takahashi et al., 2007).

The olfactory bulb (OB) transmits smell information from the nose to the brain and is necessary for the proper sensation of smell (Wacker and Ludwig, 2012). To further determine whether the blockade of olfactory sensory input affects the PPI response, OB transmission was inhibited by a local microinjection of lidocaine or MK-801 in the OB to measure the effect on the PPI response. To better understand the neural substances that underlie the contribution of olfactory areas to the PPI response, a recombinant pseudorabies virus (PRV614) that expressed monomeric red fluorescent protein (mRFP) was simultaneously microinjected into the PPTg, which was included in a classic PPI circuit (Fendt et al., 2001); various regions were retrogradely labeled that innervated the PPTg (Banfield et al., 2003) to explore the olfactory related cortices that participated in PPI response regulation.

## Materials and Methods

### Animals

Male Sprague Dawley rats (11–12 weeks) that weighed 220–250 g were obtained from the Experimental Animal Center, Kunming Medical College, Kunming, China. The rats were housed in standard cages (4 rats per cage) at a constant temperature of 25 ± 0.5°C and 60 ± 5% relative humidity with an alternating 12 h light/12 h dark cycle (lights on at 7:00 AM). Food and water were available ad libitum. All animals were treated in accordance with the Wuhan Institute of Physics and Mathematics (WIPM), Chinese Academy of Sciences (CAS) guidelines regarding the practice of animal care. The animal facilities and experimental protocols adhered to the Association for Assessment and Accreditation of Laboratory Animal Care guidelines.

### Drugs

MK-801 (dizocilpine, (5R, 10S)-(+)-5-Methyl-10, 11-dihydro-5 H-dibenzo [a, d] cyclohepten-5, 10-imine hydrogen maleate) and lidocaine hydrochloride were purchased from Sigma (St. Louis, MO, USA) and dissolved in 0.9% saline as the vehicle. Ketamine hydrochloride, chloral hydrate, and zinc sulfate were purchased from Shanghai Pharmaceutical Group Co., Ltd (Shanghai, China). Chloral hydrate was dissolved in deionized water as the vehicle. Lemon (Sigma–Aldrich, #262600) or mint (Sigma–Aldrich, #49599) essences were dissolved in mineral oil (Sigma–Aldrich, #91975).

### Behavioral Experiments

#### Intranasal Effusion of Zinc Sulfate

The intranasal administration of zinc sulfate (0.17 M, ZnE) and the naïve control were performed under general anesthesia (ketamine hydrochloride, 40 mg/kg, i.p.) prior to behavioral testing. Secretion and swelling in two nostrils were simultaneously induced by zinc sulfate treatment, which led to dyspnea. Two preventive measures were performed in our experiment. First, atropine was injected prior to ZnE treatment to inhibit the secretion from reducing the respiratory obstruction. Second, one of the nostrils was treated by ZnE, and 2 h later, the second nostril was treated by ZnE. The animals were placed on their backs, and 50 µl of either zinc sulfate solution or saline were stretched into each naris with a blunted syringe.

#### Odor Habituation/Discrimination Test

The odor habituation/discrimination measurement was designed to measure the breadth times of cross-habituation among different odorants and specify the degree of spontaneous discrimination between different odorants. The test was performed in a compartment (L × W × H: 0.75 × 0.5 × 0.5 m) with two glass plates placed 15 cm apart in the diagonal position. An odor (10–6v/v, lemon or mint essence dissolved in mineral oil) was introduced on one side of the compartment, and a control odor (mineral oil) was introduced on the other side. The animals were acclimatized to the compartment once per day for 5 min on three consecutive days (between 09:00 and 15:00 each day) prior to behavioral testing. On the fourth day, the habituation-discrimination behaviors were recorded. The odor and control solutions (25 µl each) were applied to filter paper and placed on the glass plate. During the trials, the animal was first presented with the lemon odor on one side of the compartment and the control odor on the other side in five consecutive 5-min trials; each trial was separated by a 15 min break interval after acclimation. In the sixth trial, lemon was replaced by a novel odor (mint), and the animals were subsequently exposed to the mint odor for 5 min. The amount of time that the animal spent investigating (sniffing) the mint or mineral oil was recorded. Sniffing was defined as when the animal placed its nose within 1 cm of the glass plate surface. During each experimental break interval, the compartment was cleaned with 75% ethanol. Odor discrimination was considered to be impaired if the animal spent less time investigating the new (mint) odor in the sixth trial.

#### Locomotion in an Open Field

The open-field apparatus was a square plywood arena with a smooth and waterproof surface (high: 35 cm, width: 50 cm). The floor of the arena was divided into 20 equal units with black lines; there were 9 central units and 11 edge units. The apparatus was lighted from above with a 40-w light bulb. The animals were habituated to the apparatus for 30 min on three consecutive days. The animals were subsequently placed in the center of the open field and were allowed to move freely for 5 min. During each experimental interval (10 min), the compartment was cleaned with 75% ethanol to clear the scent mark. The behavior and movement of each animal were recorded with a video camera positioned above the open field, and the total locomotion and time spent in the central area were measured on a computer using infrared tracing software. All open-field tests were conducted between 9:00 and 15:00 h.

#### Prepulse Inhibition Measurement Procedure and Apparatus

The acoustic startle response and prepulse inhibition were measured according to a previously described procedure (Chen et al., 2010; Meng et al., 2010). All animals were housed in a sound-attenuated room with a 60-dB ambient noise level. During the experiments, the rats were placed in a plexiglass cylinder (10 cm in diameter and 28 cm in length) in a four-unit automated ASR testing instrument (Yilike Co. LTD., Kunming, China) and were exposed to 65 dB of SPL white background noise. The small plexiglass cylinder in each of the four units was fixed to a platform; a sensitive sensor was attached underneath the platform and was connected to a PC in an adjacent room to collect the startle-response data.

The rats were provided an acclimation period prior to the test session. During the test session, the rats were placed in the test cylinder for a 5-min period, and ten trials of randomly delivered 115-dB SPL pulses or prepulsed startle stimuli were presented. After the acclimation period, the rats were exposed to 50 trials of randomly delivered acoustic stimuli delivered through a speaker above the cylinder. The acoustic stimuli consisted of 10 trials of a 115-dB SPL pulse stimulus, 10 trials without a delivered stimulus (NOSTIM) and 30 trials of a prepulsed startle stimulus. The 115 dB SPL pulse consisted of a single 20 ms sound presentation. The prepulsed startle stimuli consisted of 100 ms of 20-ms white-noise pulses that contained non-startling stimuli of 5, 10 or 15 dB SPL above the 65 dB SPL background noise (PPI 5, PPI 10 and PPI 15, respectively) followed by a single 20 ms 115-dB SPL pulse. Inter-trial intervals (ITI) of 27–32 s were used between the stimuli presentations.

### Experimental Design

All rats underwent behavioral tests 2 weeks after arrival to acclimate them to the testing environment. For the rats that underwent stereotaxic surgery (Experiments 2), the tests occurred 1 week after surgical recovery. All independent experiments were completed in independent animals, and the number of each group was 7–10.

#### ZnE Treatment Effects on the Odor Habituation/Discrimination and Locomotion in the Open Field

In Experiment 1, the rats were randomly assigned to naïve control and ZnE groups (*n* = 7–10). All treatments and tests were conducted similar to the description in ***Behavioral experiments***. To determine whether ZnE treatment impaired the rats’ olfactory sensory, an odor habituation/discrimination test was used 24 h after the ZnE or naïve control treatment. The locomotor activity was subsequently measured after the odor habituation/discrimination measurement to reduce the possibility that ZnE treatment may impair locomotion to disturb the PPI response. Following the completion of the olfactory and locomotion tests, the ZnE/naïve rats were measured for the PPI response.

#### Effects of a Lidocaine/MK-801 Microinjection into the Olfactory Bulb on the PPI Response

To confirm the hypothesis that the blockage of the olfactory sensory impaired the PPI response, the activity of the OB in the rats was selectively inactivated/blocked by lidocaine/MK-801. The rats were anesthetized with chloral hydrate (10%, 0.4 ml/100 g) 1 week prior to the experiments. A pair of stainless steel guide cannula (26G, 0.4 mm i.d., 0.5 mm o.d.) were stereotaxically implanted bilaterally into the OB (±1.5 mm lateral to the midline, −7.08 mm anterior to Bregma, and 4 mm dorsoventral to the dura) and mounted onto the skull with dental cement. A guide cannula was maintained in place throughout the experiment. On the eighth day, an infusion cannula was inserted through the guide cannula until it protruded 1 mm beyond the respective inner end.

Microinjections into the OB were performed using two needles (33-gauge, RWD Life Science, Shenzhen, China), which were connected to a 5 L syringe (RWD Life Science, Shenzhen, China) by PE-10 tubing (RWD Life Science, Shenzhen, China). A multi-channel syringe pump (RWD Life Science, Shenzhen, China) was used to bilaterally inject the agents into the OB at the same time at a rate of 0.2 µl/min for 10 min (final volume: 2 µl per side). In all animals, the infusion cannula was maintained in place for 10 min to enable adequate diffusion of the solution. This lidocaine volume has been effectively used to reversibly block cortical activity for approximately 40 min (Tehovnik and Sommer, 1997). Immediately following the lidocaine/MK-801 infusion, the animals were subjected to the PPI test. After the PPI test, the location of the infusion cannula was assessed in the post-mortem brains. The methods have been previously described (Contreras et al., 2007). The animals were sacrificed by decapitation, and the brains were removed, frozen and cut into coronal sections. The path of the infusion cannula was examined to verify that the cannula location was within the selected area of the OB.

#### Viral Transsynaptic Tracing from the PPTg in Rats

To further explore the relation between the olfactory-related cortex and PPI circuits, independent animals were administered a stereotactic microinjection of a recombinant PRV (PRV614) that expressed the mRFP into the PPTg to retroactively trace the organizational circuitry of the olfactory system that may modulate the PPI response. A detailed procedure of the microinjection has been previously described. Briefly, the animals were anesthetized with chloral hydrate (0.75 mg/kg) and then mounted in a stereotaxic apparatus. A hole was drilled on the skull above the PPTg (1.32 mm anteroposterior, ±1.90 mm mediolateral from the interaural, and 9 mm dorsoventral to the dura). Injections were performed with a glass micropipette (tip diameter—10–15 um) connected to a 10 µl Hamilton syringe. A recombinant PRV that expressed mRFP was controlled by the cytomegalovirus promoter. The PRV614 (1 × 10^8^ pfu/ml) was mixed with 0.17 µm green fluorescent beads (1:100 diluted, P7220). Then, 200 nl of the mixture were injected into the PPTg at a rate of 20 nl/min. After the injection, the glass micropipette was maintained in the brain for an additional 15 min to prevent the viral solution from diffusing away from the inject site. Four days after the injection, the rats were deeply anesthetized with 10% urethane and transcardially perfused with 500 ml of physiological saline followed by 500 ml of 4% paraformaldehyde (PFA) solution. The brain was dissected and subsequently post-fixed in 4% PFA at 4°C overnight. Coronal brain sections (40–50 µm) were sequentially prepared on a Leica VT1000S vibrating microtome and transferred into 24-well plates. The sections were subsequently wet mounted on slides with the Vecta-Shield mounting medium and were visualized with wide-field upright fluorescence microscopy (Olympus BX51). The images were further manipulated for brightness and contrast, and a montage was created using Adobe Photoshop version CS2. Scale bars were added with image pro plus (IPP 6.0).

#### C-Fos Expression in PPI-Sensitive Brain Fields by Viral Transsynaptic Tracing

To identify whether PPI-sensitive fields by viral transsynaptic tracing were a part of the neuroanatomical substrates in the PPI response, c-Fos expression in the PRV tracing-signal positive fields was confirmed by immunohistochemistry (IHC). For this experiment, independent animals were needed for treatment by different stimuli, including the control (no stimulus), PPI and Pre stimulus. After the stimulus, c-Fos immunostaining in the PRV tracing-signal positive fields was performed as previously described (Arime et al., 2012). c-Fos expression was measured to occur from 1–4 h after an acute stimulation. To confirm the fields activated by PPI stimulation, the animals were deeply anesthetized with chloral hydrate (450 mg/kg, i.p., Shanghai Chemical Reagent Co., LTD, China) 2 h after the PPI stimulus. The rats were then transcardially perfused with 0.9% saline followed by a fixative that comprised 4% PFA in 0.01 M phosphate-buffered saline (PBS, pH = 7.4). The brains were removed followed by locate in 10, 20 and 30% sucrose-0.01 M PBS to completely dehydrate the tissues. The brains were then frozen-sectioned coronally at 20 µm thickness. After permeabilization and blocking, the frozen coronal sections in the sensitive brain fields were incubated with rabbit anti-c-Fos antibody (1:200; sc-52, Santa Cruz Biotechnology) overnight at 4°C. They were washed with 0.01 M PBS buffer and incubated with biotinylated goat anti-rabbit antibody (1:200; vector Lab, CA, USA) for 20 min in 37°C. The streptovidin-conjugated horse radish peroxidase was then added, with 3, 39-diaminobenzidine (DAB) used as the chromagen (Vectastain Elite ABC Kit, Vector Laboratories, CA, USA). The sections were dehydrated through a graded ethanol series and coverslipped with resinene. The sections were examined under a 40× objective lens magnification, and c-Fos immunoreactivity (CMR) was measured with Image-Pro Plus software (Media Cybernetics, Silver Spring, USA). Statistical analyses were conducted with the SPSS statistical package.

### Statistical Analyses

The data are presented as the mean ± standard error of the mean. The percentage of the PPI was calculated using the following equation: [(startle response to a 115-dB SPL startle stimulus—response to pulses with a prepulse)/startle response to a 115-dB SPL startle stimulus × 100]. The data were analyzed for variables of interest using the statistical package for the social sciences (SPSS v. 13.0). A paired-samples Student’s *t*-test was used for odor and locomotor evaluations. The c-Fos data were analyzed using one-way ANOVA to identify group differences. The alpha level for significance was set at 0.05.

## Results

### ZnE Treatment on the Odor Habituation/Discrimination

**Figure 1:**
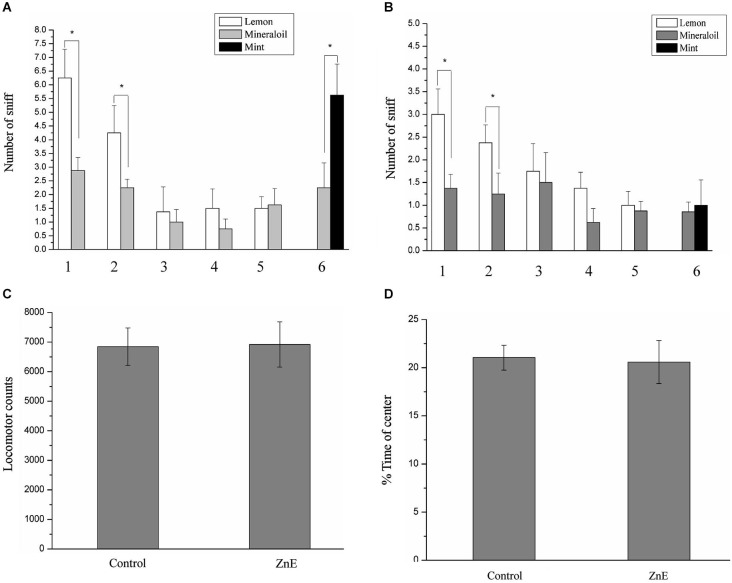
**The performance of ZnE-treated rats in a habituation/discrimination task and locomotion in an open field. (A,B)** Odor habituation/ discrimination task. Values represent the mean ± SD (*n* = 8–10). * indicates *p* < 0.05. **(C)** Effect of ZnE treatment on the locomotor activity of rats in the open field. The rats were allowed to freely explore the open field for 5 min (mean ± SEM). There was no significant difference between the groups (*F*_(2,21)_ = 0.005, *p* > 0.05). **(D)** The effect of ZnE treatment on the time spent in the central area of the open field. No differences were identified between the groups with respect to the time spent in the central area of the open field (*F*_(2,21)_ = 0.897, *p* > 0.05) compared with the naïve control group.

Our primary goal was to test whether there is a causal relationship between PPI response impairment and olfactory deficiency. We first examined odor-guided behavior in an odor habituation/discrimination task, which enabled measurements of novel odor investigation, odor learning and memory (habituation), and odor discrimination within a single behavioral test (Yang and Crawley, 2009). In this test, the rats were presented with lemon and a control odor during five consecutive days and subsequently exposed to a novel odor (mint odor, see Section Materials and Methods). Analysis of variance, using the five habituation trial numbers as the within-subject factor and the experimental group as the between-subjects factor, identified significant main effects (*F*_(4,160)_ = 50.869, *p* < 0.05) and interaction effects (*F*_(20,160)_ = 2.015, *p* < 0.05). This finding indicated that habituation to the lemon odor progressed across successive trials and was affected by the different treatment methods. *Post hoc* analysis indicated that in the odor habituation experiments (Figures 1A,B), the animals in the naïve control and ZnE groups exhibited a significant decrease in the sniffing time from the first odor (lemon) presentation through the subsequent presentations of the same odor. This decrease in sniffing behavior suggested that the animals successfully habituated to the repeated lemon odor presentations. When the lemon odor was replaced by a novel odor (mint) in the discrimination test, pairwise comparisons indicated that the control animals spent more time investigating the mint odor than the mineral oil (*p* < 0.05), whereas the animals that received the ZnE application to the naris did not exhibit a significant difference in their sniffing times between the mint and control odors (Figures 1A,B). These results suggested that the animals that received the ZnE application exhibited impaired discrimination between familiar and novel odorants (*p* > 0.05).

### Effects of ZnE on Locomotion in an Open Field

There is a possibility that locomotor activity was disturbed by ZnE to affect the PPI response. To test this possibility, we examined the locomotor activity of the rats treated by ZnE treatment. Figures 1C,D shows that there were no significant differences in total locomotion between the naïve control and ZnE groups (*p* > 0.05). In addition, no difference was identified in the time spent in the central area of the open field (*p* > 0.05) for either group. These findings indicated that no gross motor dysfunction was caused by ZnE.

### Effects of ZnE Treatment on the ASR and PPI

**Figure 2:**
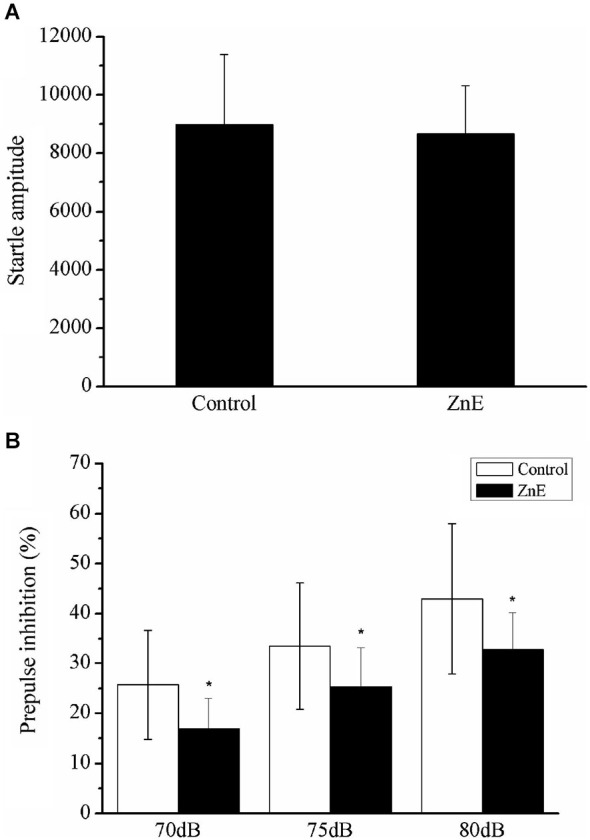
**The effects of ZnE on the startle magnitude and PPI response. (A)** The effect of ZnE treatment on the startle response magnitude. An independent sample t test identified a significant difference between the groups (*F*_(1,21)_ = 10.044, *p* > 0.05). **(B)** The effect of ZnE on the percentage change induced by a prepulse prior to an ASR PPI. A two-way ANOVA identified a main treatment effect (*F*_(2,42)_ = 4.836, *p* < 0.05), but there was no significant interaction (intensity × treatment) (*F*_(4,42)_ = 1.689, *p* > 0.05). A *post hoc* (LSD) test indicated that the ZnE treatment significantly altered the PPI response (naïve control vs. ZnE, *p* < 0.05). The data are presented as the mean ± SD. * indicates a significant difference compared with the naïve control group (*p* < 0.05).

Figure 2 shows the effect of a prepulse on the acoustic startle reflex in the ZnE and naïve control rats. The animals treated with ZnE were evaluated for their ASR and their prepulse inhibition to determine if olfaction has an impact on PPI and ASR. ZnE treatment caused significant changes in the animal’s PPI, but the ASR was unclear. The effects of ZnE treatments on the PPI and ASR are illustrated in Figure 2. An independent samples *t*-test of the ASR indicated that there was no significant difference between the groups (Figure 2A; *p* > 0.05). A two-way ANOVA of the PPI test identified a main treatment effect (*F*_(2,42)_ = 4.836, *p* < 0.05), but there was no significant interaction between the groups and stimulus intensities (Figure 2B; *F*_(4,42)_ = 1.689, *p* > 0.05). A *post hoc* analysis (LSD) indicated that ZnE treatment decreased the PPI (*p* < 0.05: naïve control vs. ZnE). These results indicated that ZnE treatment significantly reduced the PPI at all auditory intensities tested. Further analysis of the relationship between ZnE treatment and prepulse intensity was used to examine and differentiate the main effects caused by increasing the auditory intensity of the prepulse. Significant differences between the ZnE treatment and naïve control groups were identified at 70, 75 and 80 dB (Figure 2B). ZnE treatment disrupted the PPI expression in the 75 and 80 dB tests and may have led to a decrease in the startle response after an acoustic prepulse compared with the naïve control group (one way ANOVA, *F*_(2,21)_ = 5.379, *p* < 0.05; *F*_(2,21)_ = 4.820, *p* < 0.05).

### Effects of Lidocaine/MK801 Microinjections into the Olfactory Bulb (OB) on the PPI

**Figure 3:**
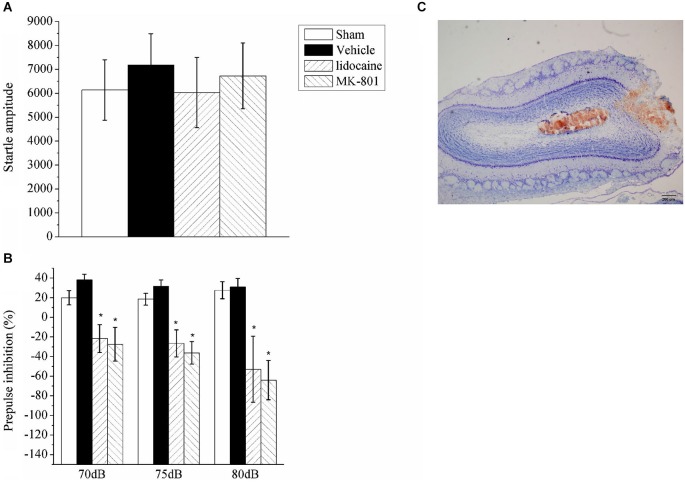
**The effect of lidocaine or MK801 bilateral microinjections into the OB on the ASR and PPI. (A)** Acoustic startle amplitude of the rats administered bilateral microinjections of lidocaine or MK801 in the OB. A one-way ANOVA indicated there was no significant difference between the groups. **(B)** The PPI (%) of the rats with the OB inhibited with lidocaine or MK801 at three different auditory prepulse intensities (70, 75 and 80 dB). A repeated measures ANOVA of the prepulse data identified significant effects of drug treatment (*F*_(3,20)_ = 5.74, *p* < 0.05). The values indicate the mean ± SD (*n* = 6). * indicates a significant difference compared with the vehicle group (*p* < 0.05). **(C)** A representative photograph of the OB that indicates the microinjection site.

In the previous section, the nasal epithelium was directly stimulated; thus, the effects identified could be mediated by these effects rather than olfaction. To further test the role of olfactory neurons in PPI, we examined the PPI response after the inactivation of OB neurons with a stereotaxic injection of either lidocaine or MK-801. Bilateral microinjections were administered in the OB of four groups of rats, including the lidocaine (*n* = 6), MK801 (*n* = 6), vehicle (*n* = 6) or sham surgery (*n* = 6) groups; the animals were then subjected to the PPI test. A repeated measures ANOVA of the data identified significant effects for drug treatment (*F*_(3,20)_ = 5.74, *p* < 0.05) and the intensity of the auditory prepulse (*F*_(2,40)_ = 1.007, *p* > 0.05), but not for the interaction between drug treatment and prepulse intensity (*F*_(6,40)_ = 0.886, *p* > 0.05). Inactivation of the OB neurons by lidocaine or MK801 injection had no effect on the startle amplitude (Figure 3A), but it significantly enhanced the startle response following the prepulse (Figure 3B). The injection sites in the OB were identified using Nissl staining (Figure 3C).

### Regions in the Olfactory Cortices Give Rise To Afferent Circuitry to the PPTg

**Figure 4:**
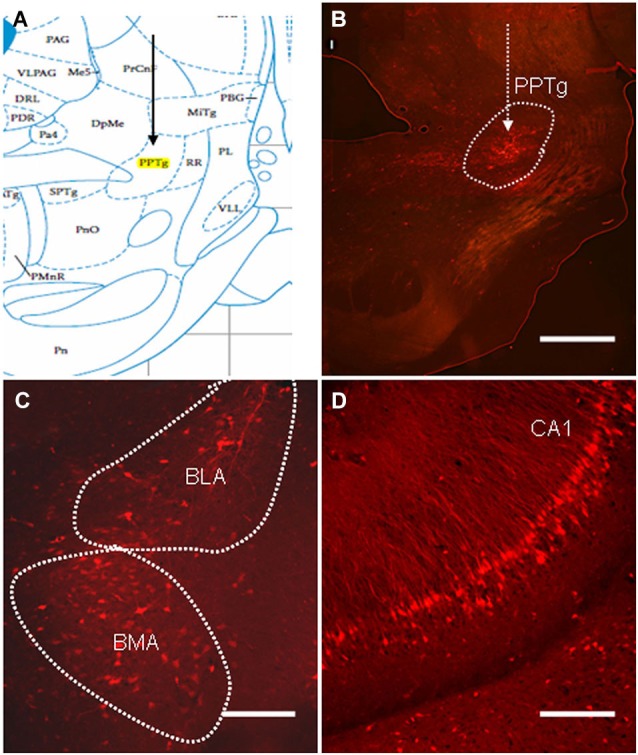
**The distribution of PRV614-mRFP trans-synaptically labeled neurons in regions known to mediate PPI after 4 days of infection. (A)** A schematic map of the midbrain (PPTg is highlighted in yellow). **(B)** PRV614-mRFP labeled neuron distribution is restricted to the PPTg near the injected site. **(C)** Trans-synaptically labeled neurons in the BLA and the basomedia amygdala (BMA). **(D)** Trans-synaptically labeled neurons specifically distributed in the CA1 of the ventral hippocampus. Scale bar in **B** = 1 mm; scale bars in **C** and **D** = 200 µm.

Although the PPTg has been shown to play a critical role in mediating the PPI of the ASR (Takahashi et al., 2007), the exact role of olfactory contribution to the PPI response is unclear. Retrograde viral tracing was used to investigate whether neurons in the olfactory cortices directly participated in the regulation of the PPI of the ASR. The virus PRV614, which expressed mRFP, was injected into the PPTg and allowed to spread retrogradely for 4 days (Figure 4A). On the fourth day, the rats exhibited moderate signs of viral infection. At this point, the infected rats were anesthetized with 20% urethane, perfused transcardially with PFA, and the location of viral expression was examined with fluorescence microscopy. Labeled neurons were identified near the targeted injection site and were specifically restricted to the PPTg (Figure 4B). Several regions that have been shown to regulate the PPI of the ASR were retrogradely labeled in the ipsilateral hemisphere. The most prominently labeled regions were the basolateral amygdala (BLA), basomedial amygdala (BMA) and CA1 of the ventral hippocampus (Figures 4C,D). These results indicated that the PRV614-mRFP trans-synaptic tracing successfully identified areas found to regulate the PPI of the ASR, which is consistent with the areas identified using lesion, electrophysiological and pharmacological approaches (Contreras et al., 2007).

**Figure 5:**
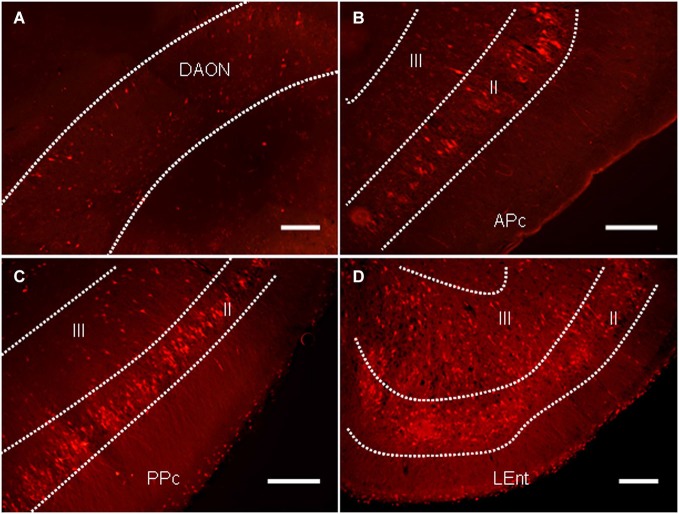
**The distribution of PRV614-mRFP trans-synaptically labeled neurons in several regions of the olfactory cortices after 4 days of infection. (A)** The distribution of trans-synaptically labeled neurons in the dorsal aspect of the anterior olfactory nucleus (DAON). **(B)** The distribution of trans-synaptically labeled neurons in the anterior piriform cortex (APc). **(C)** The distribution of trans-synaptically labeled neurons in the posterior piriform cortex (PPc). **(D)** The distribution of trans-synaptically labeled neurons in the lateral entorhinal cortex (LEnt). Scale bar = 200 µm.

**Figure 6:**
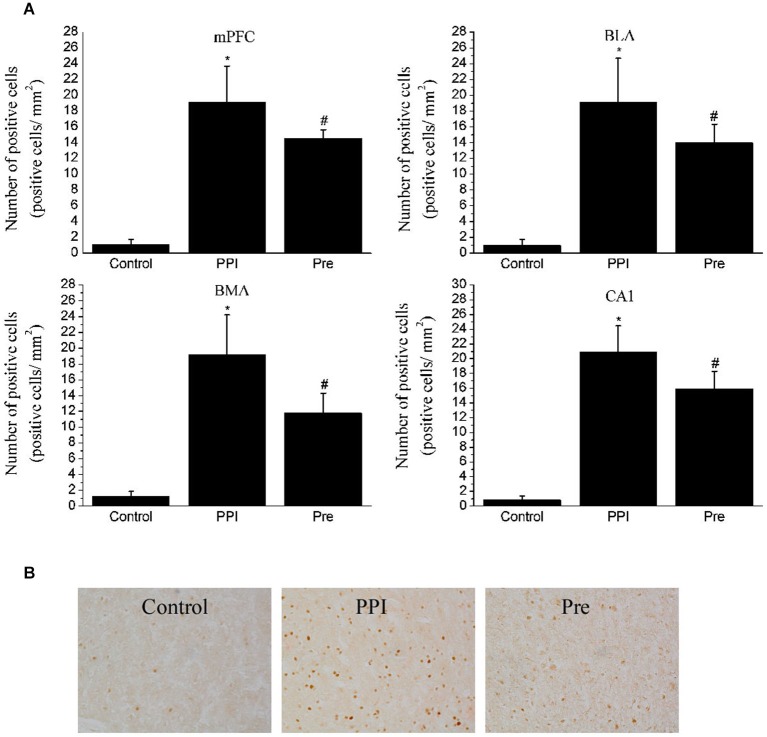
**Expressions of c-Fos in selected areas of the rats in the naïve control, PPI and prepulse groups. (A)** One-way ANOVA analysis: mPFC, *F*_(2,12)_ = 58.17, *p* < 0.05; BLA, *F*_(2,12)_ = 34.95, *p* < 0.05; BMA, *F*_(2,12)_ = 38.84, *p* < 0.05; CA1, *F*_(2,12)_ = 87.92, *p* < 0.05. * indicates *p* < 0.05 vs. the naïve control group and # indicates *p* < 0.05 vs. the PPI group. Values indicate the mean ± SD (*n* = 4). **(B)** Representative photomicrographs of c-Fos staining in the mPFC are shown (20X).

In addition to the regions known to regulate the PPI of the ASR, a large number of labeled neurons were distributed in several areas of the primary olfactory cortex, including the anterior olfactory nucleus (AON), the piriform cortex (Pcx) and the lateral entorhinal cortex (LEnt; Figure 5). The majority of the labeled neurons in the AON were distributed in the dorsal aspect (Figure 6A). Most labeled neurons in the Pcx were identified in layer II of the Pcx and were distributed from the anterior (APc) to posterior (PPc) aspects. The neurons in layer III of the Pcx were also labeled to some extent (Figures 5B,C). The neurons in the LEnt were prominently labeled in both layers II and III (Figure 5D).

### C-Fos Expression

To verify that the regions identified with viral transsynaptic tracing are a part of the neuroanatomical structure that participates in the PPI response, we examined c-Fos protein expression in the mPFC, amygdala and hippocampus fields in rat brains from the naïve control (*n* = 4), PPI (*n* = 4) and Pre (*n* = 4) groups using an IHC method. After the PPI and Pre stimulus, a significant difference in c-Fos expression was identified in the mPFC (*F*_(2,12)_ = 58.17, *p* < 0.05), BLA (*F*_(2,12)_ = 34.95, *p* < 0.05), BMA (*F*_(2,12)_ = 38.84, *p* < 0.05) and CA1 (*F*_(2,12)_ = 87.92, *p* < 0.05). Representative photomicrographs of c-Fos staining in the mPFC are shown in Figure 6.

## Discussion

In the present study, to determine whether blockage of olfactory sensory input induced by zinc sulfate infusion in the olfactory naris (0.5 ml, 0.17 M, ZnE) can disturb the PPI response, we performed behavioral and pharmacological tests in rats, including an odor habituation/discrimination test, locomotion test, IHC, and virus tracing. Our results demonstrated that blockage of olfactory sensory input by ZnE treatment impaired the PPI response with no effect on locomotion and inhibited the expression of c-Fos in PPI-related sensitive fields. Importantly, the reversible blockade of activity in the OB by lidocaine or MK-801 also impaired the PPI response. In addition, PRV614 tracing studies demonstrated that many fields in the brain were identified between the olfaction-related cortex and PPI-related brain areas (PTTg area) (Fendt et al., 2001). Thus, it is highly likely that olfactory impairment can disturb the function of the PPI system. More importantly, as part of forebrain structures, this study reveals that the olfactory sensory input contribute to regulate the PPI response by mean of top-down.

Previous studies have demonstrated that ZnE impaired the sensory neurons in the olfactory epithelium and widely disturbed olfactory sensory and perception functions (Niu et al., 2013). For example, ZnE treated rats, in this study and previous studies (Meng et al., 2010), exhibited disabilities in investigation, discrimination and habituation in response to various odors. In addition to the assessment of olfactory function in ZnE-treated rats, the locomotor activity in the animals was also assessed in this study to exclude possible anxiety related effects and depressive behaviors associated with excessive ZnE treatments, which may have resulted in confounding issues that affected the PPI response (Ahmari et al., 2012). Previous studies have indicated that bulbectomy may have caused anxiety or depression, as well as PPI impairment (Bilkei-Gorzo et al., 2002). However, our results indicated that the impairment of olfactory perception could disturb the PPI response with no effect on locomotor. Namely, the results are based on the olfactory lesion but not locomotor impairment.

Furthermore, OB played a vital role in olfactory relaying from environmental stimulus to sensory cortex (Kay and Sherman, 2007). From the anatomy, olfactory cortex was parts of forebrain structure, which projected to subcortical structures including the PPC and amygdala.

Olfactory cortex stimulation altered the forebrain reward and motivational behavior. The olfaction-driven behaviors required an intact olfactory system to detect and discriminate the environmental stimulus from background stimuli (Fitzgerald et al., 2014). At the same time, the abilities of relaying the olfactory information into forebrain structures including emotional and reward-related brain structures were required in top-down pattern modulating (Wilson and Mainen, 2006; Wilson and Sullivan, 2011; Fitzgerald et al., 2014).

The olfactory system would interact with these forebrain structures that were involved in modulating the primary PPI pathway and then regulated these brain fields’ activities by the mean of top-down (Li et al., 2009; Du et al., 2010, 2011; Ma and Luo, 2012). Although the primary neural pathways mediating PPI response were located in the brainstem (Davis and Gendelman, 1977; Li and Frost, 2000; Fendt et al., 2001), the forebrains were included in primary neural pathways including PPTg and were top-down modulated. For example the primary auditory cortex, lateral nucleus of the amygdala and posterior parietal cortex also played a role in the top-down modulation of PPI in rats (Bakshi and Geyer, 1998; Miller et al., 2010; Du et al., 2011). At the same time; some higher-order cognitive processes could modulate the PPI response (Neumann, 2007; Du et al., 2010). Some evidences confirmed that directing attention to the prepulse stimulus could enhance PPI response (Heekeren et al., 2004; Annic et al., 2014). In patients, attentive deficit was found in anosmia cases (Roberts et al., 2010; Garcia-Sanchez et al., 2011). Olfactory sensory stimulus affected on the attentional response by top-down regulations via descending axonal projections from forebrain structures (Georgoussi et al., 1990; Yochman et al., 2013). So we concluded that olfactory sensory stimulus modulated the PPI response by affecting the attention.

To further examine the role of olfactory sensory input and its disruption of PPI, the rats were transiently microinjected with lidocaine in the OB. The OB is the first relay station that transmits olfactory sensory information to the higher olfactory cortex (Lledo et al., 2005). The PPI was similarly reduced in the lidocaine treated and ZnE treated rats. Furthermore, MK801, an N-methyl-D-aspartate (NMDA) receptor antagonist (Lledo et al., 2005), injection into the OB also reduced the PPI response. These results suggested that glutaminergic neurons in the OB may have played a regulatory role in the startle reflex and possibly the PPI phenomenon. The reduced PPI effect was dependent on olfactory specific locations: limiting olfactory stimulus by both ZnE in the nasal epithelium and lidocaine blockage of the OB resulted in an impaired PPI phenomenon (Figures 2, 3). These results emphasized the fact that an olfactory stimulus plays an important role in the regulation of the PPI response.

Although these experiments demonstrated that blockage of olfactory sensory input could impair the PPI response, the circuit basis that the olfactory system interacted with the PPI system was not identified. Previously, a retrograde transsynaptic tracer (PRV) was used to identify the CNS regions that innervated the target areas (Rodriguez-Sierra and Terasawa, 1979). Thus, the PRV614 retrograde tracing from the PPTg was used to demonstrate that circuits existed between the olfactory systems in the PPTg, which may provide a potential neural circuit for olfaction to regulate the PPI response. Our data demonstrated PRV614-mRFP trans-synaptically labeled neurons were present in several regions of the olfactory cortices, including the dorsal aspect of the anterior olfactory, APc, PPc, and lateral entorhinal cortex (LEnt), after 4 days of PRV injection. Simultaneously, the labeled neurons of classic PPI networks, such as the mPFC, BLA, BMA and CA1 of the hippocampus, were also identified after 4 days of PRV injection (Swerdlow et al., 2001). The results demonstrated that the olfactory system was indirectly connected with the PPI fields. Furthermore, olfactory information was first transmitted through the OB to the limbic circuitry and then relayed to PPI-mediating regulatory circuitry in the brainstem.

Previous studies have demonstrated that the neural circuits that included the PPI pathways can be labeled by c-Fos mapping. This method is suitable and applicable to detect neurons activated by various stimuli (Knapska et al., 2012). Our data indicated these fields traced by PRV were also confirmed by c-Fos expression. Studies have demonstrated the mPFC, BLA, BMA and CA1 of the hippocampus were included in the regulation of PPI (Schwabe et al., 2004; Stevenson and Gratton, 2004).

In conclusion, ZnE-treated rats exhibited normal locomotor activity but an impaired PPI to a startle reflex, which suggested that olfactory dysfunction played a role in sensorimotor gating, but not in overall physiological functions. PRV retrograde tracing from the PPTg identified important circuitry interactions between the olfactory system and other neural circuits that regulate PPI. Although olfactory dysfunction has not been commonly used by clinicians, the results presented here highlight a connection between olfactory function and psychiatric illness.

## Author Contributions

HN, QZ and XH have bred and maintained the animal colonies and litter details including litter size and litter survival within this study. HN and TZ performed the behavior test and IHC experiments. TZ and XS performed blind data scoring for c-Fos test. ZZ and XH performed the PRV tracing experiment. YQ, FX and MH conceived the study and participated in its design and coordination and drafted the manuscript. All authors read, edited and approved the final manuscript.

## Conflict of Interest Statement

The authors declare that the research was conducted in the absence of any commercial or financial relationships that could be construed as a potential conflict of interest.
